# Artificial Respiration

**Published:** 1878-12

**Authors:** S. B. Chase

**Affiliations:** Osage, Iowa; Medical and Surgical Reporter


### Artificial Respiration.

In the absence of myself and partner, Dr. Whitley, an earnest message came, Friday, P. M., December 21st, 1877, to call at the residence of Mrs. S., an American, about forty-two years of age. On returning, Dr. W. answered the summons, but found Dr. M. in attendance. The latter had just administered morph. sulph., for nervous excitation, in part the result of some family trouble, therefore the case was left in charge of Dr. M.

In about an hour Dr. W. was re-summoned. Returning, he found the patient cold, comatose, unable tó swallow, and apparently dying. She drew her last gasping breath as he re-entered her room. Artificial respiration was at once resorted to. A subcutaneous injection of atropia sulph. was administered, cold applied to the head, and heat, stimulants of friction to the body and extremities. I was then called in consultation.

The atropia was repeated, and the general treatment continued. In about an hour the pupils began to dilate, and the body to regain slight warmth; yet no other signs of returning life appeared. There was no pulse at the wrist; and if artificial respiration was momentarily suspended, the heart's tremulous motion ceased. Much of the time the tongue was drawn forward and held; otherwise no air could be forced into the lungs.

This treatment was energetically continued, with alternating hopes and fears, about seven hours. Feeble, unaided respiration was then resumed, and maintained about half an hour. It then ceased, and artificial was again resorted to. After about the same length of time she again began "to spin the brittle thread of life," and was able to swallow.

Partial consciousness returned Saturday morning, and hopes of recovery were entertained during the day, and a portion of the following. In defiance of precautionary efforts, however, pneumonic congestion came on Sunday afternoon, the patient relapsed into a profound coma, and died Monday evening, about seventy hours after taking the morphia.

The remarkable features in this case were the length of time during which artificial respiration wes maintained, and the small amount of morphia; just one-sixth of a grain, alleged to have been given. The patient was previously healthy.

Osage, Iowa. S. B. CHASE, M. D.

*Medical and Surgical Reporter.*

